# Development and Improvement of an Effective Method for Air and Surfaces Disinfection with Ozone Gas as a Decontaminating Agent

**DOI:** 10.3390/medicina56110578

**Published:** 2020-10-30

**Authors:** Giuseppina Moccia, Francesco De Caro, Concetta Pironti, Giovanni Boccia, Mario Capunzo, Anna Borrelli, Oriana Motta

**Affiliations:** 1 Department of Medicine Surgery and Dentistry “Scuola Medica Salernitana”, University of Salerno, via S. Allende 1, 84081 Baronissi (SA), Italy; gmoccia@unisa.it (G.M.); fdecaro@unisa.it (F.D.C.); gboccia@unisa.it (G.B.); mcapunzo@unisa.it (M.C.); omotta@unisa.it (O.M.); 2 University Hospital “San Giovanni di Dio e Ruggi D’Aragona”, via S. Leonardo, 1, 84131 Salerno, Italy; direzione.sanitaria@sangiovannieruggi.it

**Keywords:** sanitization, public health, human health, ozone, air treatment, contamination

## Abstract

*Background and objectives:* Ozone has been one of the most investigated and discussed sanitization methods. This paper reports a procedure to sanitize air hospital environments, in particular chirurgical surgery rooms that require high levels of disinfection. The purpose of this work was the development and implementation of a cleansing and sanitizing procedure for critical clinical settings with ozone, to prevent hospital infections by the elimination of all toxic and harmful microorganisms in the air, and ensure safe use for operators and patients. *Materials and Methods:* The protocol for the study involved a structured selection of a representative environment of healthcare structures such as high, medium, and low-risk settings in air and examples of hospital furniture. *Results:* The concentration of ozone was measured during sanitization treatment and the estimation of the total microbial count in the air and on different surfaces before and after the sanitization operations was performed. The results demonstrated a significant reduction in the microbial count that always fell below the threshold value. *Conclusions:* Currently, there are no air treatment strategies available for inactivating airborne organisms during hospital outbreaks, which is most probably due to the lack of approved protocols.

## 1. Introduction

During the recent world public emergency, the healthcare system was involved in the healthcare response to face the pandemic with new strategies. In most instances, microorganisms are believed to be transmitted through many routes: respiratory droplets from person to person, inhalation or deposition on mucosal surfaces, by a vehicle (water, food, fomites, or inanimate objects) or a vector (insects), contact with contaminated fomites and the inhalation of aerosols produced during aerosol-generating procedures. The highest risk of healthcare-associated transmission occurs in the absence of standard precautions when basic infection prevention and control measures for respiratory infections are not in place. The critical point was the introduction of a sanitizing process minimization of virus deposition on surfaces and pathogens transmission through aerosol. In the literature, it was reported that aerosol droplets could travel short distances, even if they could persist in the air for a long time and so move over long distances (more than 1 m) [1,2,3]. Bioaerosols could also settle after a long period, leading to fomite contamination [4] and from these contaminated fomites, the further propagation of pathogens is also possible [5]. In many studies, it was reported that the airborne transmission route has been proven to facilitate the transmission of tuberculosis [6], respiratory viruses such as influenza and rhinoviruses, gastrointestinal viruses such as rotavirus [7], and is suspected of playing a role in the transmission of other pathogens such as norovirus [2]. For these reasons, strategies for the decontamination of hospital environments are described in guidelines proposed by various international committees, in particular, the advisory committee for the practice of controlling sanitary infection underlines the importance of correct cleaning operations to promote decontamination and the necessary use of disinfectants to reduce the microbial surface contamination in hospitals [8,9,10,11,12]. Although cleaning protocols are applied and appropriate disinfectants are used in the right concentrations, air disinfection protocols have been overlooked by scientific research and the public committee and all treatments are not sufficient to protect patients susceptible to serious and life-threatening infections [13].

In particular, cleaning and sanitizing processes in hospital environments, especially for high-risk areas such as surgery and intensive care units, are fundamental activities to ward off possible infections. The risk of infections could be reduced using appropriate environmental hygiene protocols to guarantee a low environmental microbial load.

The World Health Organization (WHO) described numerous alternatives for disinfecting surfaces, such as ethanol, isopropanol, peracetic acid, glutaraldehyde, chlorine [14,15,16], phenols, polyphenols [17], quaternary ammonium salts [18], tertiary amines, chlorhexidine gluconate, formaldehyde gas fumigation, glutaraldehyde soaking, high-pressure steam sterilization technology, and ozone (O_3_) fumigation [19,20]. The role and mechanisms of the various applied agents remain undetermined.

In 1982, ozone was recognized as a “safe gas” to be used as a sanitizer in environments contaminated with bacteria, viruses, germs, as well as mites and insects [21]. It is also compatible with the International Organization for Standardization (ISO) and the Eco-Management and Audit Scheme (EMAS) protocols. Ozone is an excellent disinfectant thanks to its chemical characteristics to attack and oxidize all types of organic and inorganic compounds, with an antiseptic efficacy similar to fluorine. It can be used as a safe and effective agent to improve the hygienic quality of the environments, thanks to the complete removal of pollutants in the air and the inactivation of species present on surfaces difficult to reach by operators. In the literature, Ozone gas was reported as an effective compound in decontaminating FFP (Filtering Face Piece) respirators without damaging them, although it presents risks for the safety and health of workers who carry out the process if it is not handled properly [22].

A recent study of Dubuis demonstrated the efficacy of air treatment for phage and MNV-1 (eukaryotic murine norovirus) inactivation using low ozone concentrations, 1.13 ppm ± 0.26 ppm, and 0.23 ppm ± 0.03 ppm, respectively, at various relative humidity levels and exposure times of up to 70 min. An exposure of 40 min at 85% relative humidity yields the inactivation of at least two orders of magnitude for φX174, MS2 (phages) and MNV-1An. The exposure with 20% relative humidity for 10 min for other phages (PR772 and φ6) was enough to yield the same results [23].

This international overview convinced us to investigate the efficiency of ozone treatment in real use during the healthcare sanitization process and the evaluation of safety for operators and patients. Moreover, for the first time, this study also wants to underline the importance of the procedures applied to obtain good performance in the ozone sanitization process evaluating concentration levels during and after the process.

### 1.1. Environmental and Human Health Risks

Ozone gas is effective in decontamination processes without damaging surface, although it presents risks for the safety and health of workers who carry out the process if it is not properly handled. The inhalation of ozone vapors is the highest health risk since the main damages induced by this gas are mainly borne by the respiratory system; moreover, ozone is a strong oxidizing agent, which reacts violently with organic compounds such as benzene, ethylene, dienes and alkanes, therefore, it is necessary to take adequate safety measures during its use.

Ozone is a very strong oxidizing agent and it is considered one of the secondary pollutant components of photochemical smog, which produces effects on human health and property [24]. In the outdoors, ozone plays an important role in the chemistry of the atmosphere. It is produced by chemical reactions with precursors such as nitrogen dioxides NOx, volatile organic compounds VOCs, carbon monoxide CO in the presence of sunlight and it has a major role in heterogeneous reactions, which often give rise to the generation of volatile organic products [25,26].

In the literature, the ozone is well documented as an air pollutant and airway irritant, as continuous exposure to ozone induces the decrease in pulmonary function as measured by forced expiratory even in healthy children with an increase in pulmonary exacerbation frequency [27]. In vitro, human epithelial cells exposed to 1.5 ppm ozone displayed decreased cystic fibrosis transmembrane conductance regulator expression and function. Multiphase reactions of the ozone with human skin oils impact indoor air quality by depleting the ozone and forming semi-volatile organic compounds, which can be respiratory and skin irritants [28,29].

Indoor ozone concentration can vary significantly from less than 5 ppb to over 50 ppb, generate from outdoor-to-indoor transport and due to its toxic effects, many studies provide new strategies to remove ozone with improving the health conditions of people [30].

In particular environments, the use of catalytic converters was necessary to remove ozone, for example in aircraft cabins where the concentration of O_3_ can be very high, with values ranging from ~30 ppb to several hundred ppb depending on the altitude, latitude, and time of year [31].

Other researchers reported the use of ornamental plants, such as *Dracaena deremensis*, *Tagetes erecta* and *Lilium candidum*, in the remediation of the indoor ozone with the removal effectiveness in the range of 0.7–13% for the leaf surface area to room volume ratio of 0.06/m regarding an air exchange system and background loss present in an indoor environment [32].

For all these reasons, it was necessary to remark and draw up a precise protocol to be followed by operators:

- Use the ozone sanitization cycle only in the absence of people;
- Do not use in the presence of flammable substances such as alcohol, petrol, hydrocarbons, bromine, hydrobromic acid, nitrogen oxides and nitroglycerin;
- Avoid exposure to UV rays produced by fluorescent lamps;
- Seal off the doors and windows of the environments before beginning ozone generation using proper sealing gummed papers in the door and window blows.

Healthcare structures, such as hospitals, senior specialized hospitals, elderly care facilities, and postnatal care centers have occupants very susceptible to air contaminants and it necessary to plan an accurate analysis of the indoor air concentration of the ozone and its relationship to other indoor environmental factors [33]. In this work, we analyzed the concentration of gas during ozone sanitization processes to reduce health risks for operators.

### 1.2. Sanitization Procedures

Wet dusting, cleaning and the subsequent disinfection of furniture, equipment, furnishings, walls and floors, which are present in hospitals according to traditional cleaning procedures, cannot guarantee the removal of microbial agents, in particular structural ravines and the equipment or surfaces difficult to reach by the operators responsible for the sanitization and disinfection of rooms.

Sanitation in hospitals is referred to as the set of operations aimed at making a healthier determined environment according to the hygiene standards required [34,35]. Each environment, therefore, has an optimal standard that is a function of the intended use of the environment itself. For example, surgery needs a sterile condition, while sanitization may be sufficient in other hospital wards.

Ozone is produced using crown discharge generators. The air inside is moved by the recirculation fan through the machine where it comes enriched with ozone. Automatically, as described by the suppliers, at the end of the ozonation phase, the catalyzing phase begins. During the latter, the residual ozone in the air passes through the UV-C lamps, which converts ozone into oxygen eliminating any residue.

Any time the ozone sanitization is planned, the operators have to apply a precise preparation that can be resumed as follows:

- Before using the ozone generator, check the correct positioning of the supply;
- Make sure that a differential switch automatically protects the power socket upstream;
- Connect the unit to a grounded power outlet;
- Place the ozone generator in the center of the room.

Moreover, it is important to ensure having sealed off the doors and windows of the environments before beginning ozone generation, to avoid dispersion in neighbor rooms. At the end of the sanitization cycle, the machine switches off automatically while remaining powered and it is possible to remove the gummed paper from the doors and windows and store the generator in a dry environment.

## 2. Materials and Methods

In order to use measurable outcomes to investigate the hospital cleaning as a scientific process, we performed microbiological analyses in two different environments (office, and general surgery unit) according to the international standard UNI EN 13098:2002 for air sampling and UNI EN ISO 14698-1 for surface. The reference parameters to standard disinfection test according to Microbial load at 30 °C UNI EN ISO 13098:2002, UNI EN ISO 14698-1 are reported in Table 1.

Analyses were performed using 24 cm^2^ Rodac (Replicate Organism Direct Agar Contact) plate with two different substrates, PCA (plate count agar to total microbial count) and SABOURAUD DEXTROSE AGAR (to isolate mold and yeast), specific for the monitoring of environment hygiene (air and surface) to validate the cleaning and disinfection operations. For surface analyses, the contact time of plates on the surface was about 10 s to obtain uniform and constant pressure throughout the area. The plates were incubated under aerobic conditions at 30 °C for 48 h. The number of microorganisms per plate (CFU) was calculated from the number of colonies obtained on the plates containing less than 300 colonies/plate. All samples were collected before and after disinfection processes to evaluate the efficiency of the ozone cleaning protocols in the hospital wards. The protocol for the study involved a structured selection of the representative environments of a healthcare structure such as high, medium, and low-risk room in the air and examples of hospital’s furniture. For each room, 10 to 14 high-touch surfaces were chosen for the assessment of the cleanliness (door handles, furniture, bed, etc.) and the cleaning staff was not informed about the sampling. Each surgery room analyzed was about 36 m^2^, with a volume of 120 m^3^, while the office room was about 26 m^2^, with a volume of 90 m^3^. In surgical theatres, 15 air exchanges per hour with 0.24 m/s ventilation were set. The temperature was between 24 and 25 °C and the relative humidity between 40 and 50%.

Air samples were collected by SAS 180 S (SURFACE AIR SYSTEM monitoring instruments) system for microbiological environmental monitoring, used in combination with contact plates. The instrument was positioned one meter from the ozonization system, which was calibrated to start measurement after 5 min to eliminate interferences of operators in the room and sampling 1000 L of air in 6 min. The measurements were done before, during, and after treatments.

Ozone concentration was evaluated utilizing Airnova sensors, calibrated and certified by the suppliers.

## 3. Results

The performances of the ozone as a sanitation system are shown by the results of Table 2 and Table 3 that summarize the results of the swabs and air sampling carried out in healthcare facilities.

The significant reduction in the microbial count, which always falls below the threshold value, demonstrated the effectiveness of the system.

The following tables show that for the air and the analyzed surfaces such as the table, furniture, desk, etc. the ozone sanitization system succeeds to eliminate about 90% of the microorganisms present.

## 4. Discussion

**Figure 1:**
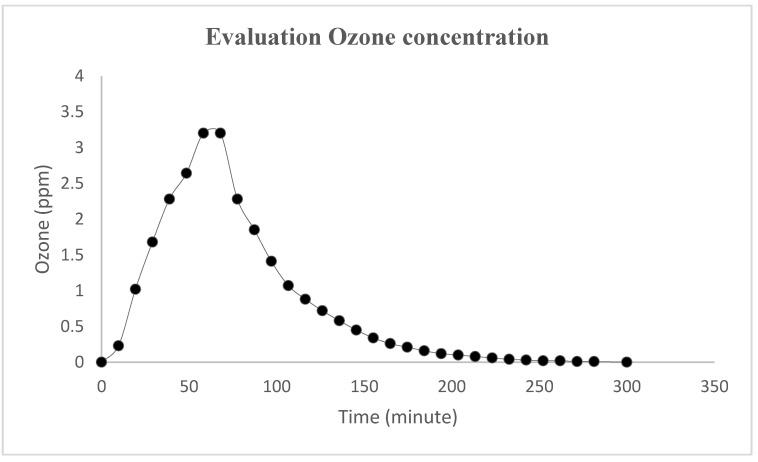
O_3_ concentration measured during treatment by the ozone sensor.

This study assessed the inactivation of airborne and surface contaminants in healthcare structures using ozone (see Figure 1). To date, no study, to our knowledge, has analyzed the correct ozone protocol for sanitization healthcare facilities with different risk levels (low, moderate, high).

Cross-contaminations, disinfection and cleaning strategies play an important role in the everyday organization of hospitals and many scientific studies are reporting precise protocols for sanitizing healthcare environments [36,37,38]. The focus of new scientific research was the introduction of sanitizing processes that avoid microorganisms’ deposition on surfaces and pathogens’ transmission through bioaerosol. In particular, international health organizations introduced more rigorous measures to optimize the quality of care provided to infected patients and to reduce the risk of pathogen transmission to other patients or healthcare operators.

Regardless of the type of surface (hands, environmental surfaces, fabrics), the objective of a cleaning and sanitizing procedure was to reduce contamination to an acceptable level of safety by applying operating methods designed to remove pathogens from air and surfaces [39,40].

In Table 2 are reported the results obtained by analyses in a staff room which is considered at low risk. Before the ozone treatment, the hygienic air performance was type C according to standard reference parameters and with a PCA value of about 100 CFU/m^3^. After treatment, the microbial count was 1 CFU/m^3^ showing a good efficiency, of more than 97%, of the ozone sanitizing procedure. In the staff room, we analyzed the furniture and white coats of medical staff, which could be also disinfected by ozone with efficiency and a total microbial load reduction of more than 90%.

Surgery units must be divided into progressively less contaminated areas, from the entrance to the surgery rooms; clothing required must be indicated by specific signs. Differentiated internal routes must be guaranteed for dirt and clean through organizational/functional and/or structural interventions that allow the safe collection and the transport of materials [41].

In Table 3 is reported the efficiency of ozone sanitization in surgery units and surgical rooms (high-risk areas) where the values were always very low to guarantee the necessary disinfection level as requested by international guidelines for hospital settings. However, in the case of very low contamination, such as 1 or 2 colonies, ozone treatment permits to obtain the complete disinfection of air and surface. The results of different units are not shown in the tables because we found the same values also in these cases, confirming good performance for the ozone process compared to other technologies reported in the literature [42].

The innovative approach of this work concerns the evaluation of airborne microbial contamination and the analysis of ozone concentration during sanitization and until the end of the process to verify and to avoid the residue of ozone itself in the room that can impair health concerns in the presence of operators. Figure 1 shows that the concentration of the ozone reaches 3.2 ppm in 1 h and completely decreases in 5 h, therefore, to completely guarantee risk prevention for the healthcare operators, the sanitization procedure was done in the evening, at the end of the working day.

In all cases, the operators will have to perform ozone sanitization after ordinary cleaning because ozone inactivates microorganisms but does not remove them and in the presence of biofilm, the process could not be efficient. This study underlines the essential role of deep cleaning before the disinfection and sterilization process to eliminate inorganic and organic materials, whose presence on instruments inhibits these processes.

## 5. Conclusions

Our objective was to develop a practical method utilizing the known anti-microbial properties of ozone to decontaminate air and surface rooms in healthcare facilities, evaluating the correct procedures focused on safety for patients and professionals.

The methodology analyzed in this work provides complete evidence on the sanitization of hospital settings, demonstrating that ozone treatment could be a useful sanitization process for hospital infection-control programs.

Ozone treatment is a very efficient method and allows raising the safety standards from the infectious point of view of health structures. There are many advantages of this device: it is easy to use, guarantees a reduction of microorganisms, and ensures the complete inactivation of airborne microorganisms avoiding the subsequent deposition on the surfaces. However, cleaning procedures with detergents is a mandatory step before any treatment to remove completely organic matter on surfaces. Despite these limitations, this study provides evidence for an ozone-based user-friendly, effective method of disinfection for critical environments such as hospitals and in particular, surgery rooms. In this work was also underlined the risk of ozone exposure for the operators and the measurements report a total reduction of ozone concentration at the end of the treatment. The evaluation of work satisfaction was done through an anonymous test. All the operators involved highlighted the important role of the training course received and the indications implemented in the protocol. Workers feel safer and more safeguarded in their daily operations.

This paper reports, for the first time, an evaluation of the ozone sanitization characteristics along with a measurement of the ozone concentration in the air being aware that the health of operators must be guaranteed likewise to that of the patients.

## Figures and Tables

**Table 1:** Reference parameters to standard disinfection test.

**Hygienic Surface Performance**	**Good**	**Satisfying**		**Unsatisfying**
CFU/plate	0–25	26–50		over 50
**Hygienic Air Performance**	**A**	**B**	**C**	**D**
CFU/m^3^	<1	10	100	200

**Table 2:** Total microbial load estimated on sampling performed in the air (CFU/m^3^) and on different surfaces (CFU/plate) in an office (results are averaged on 10 measurements).

AIR and Surface Analysed	Sabouraud Pre-Treatment	SD	Sabouraud Post Treatment	SD	(PCA) Pre-Treatent	SD	(PCA) Post Treatment	SD
AIR (CFU/m^3^)	0		0		105	±4	1	±1
DESK (CFU/plate)	10	±1	5	±1	35	±3	5	±1
TABLE (CFU/plate)	10	±2	5	±1	18	±2	2	±2
PRINTER (CFU/plate)	14	±1	7	±2	10	±1	3	±1
Air-conditioning (CFU/plate)	10	±1	7	±1	12	±2	4	±1
White coats (CFU/plate)	0		0		36	±1	1	±1

**Table 3:** Total microbial load estimated on sampling performed in the air (CFU/m^3^) and on different surfaces (CFU/plate) in a surgery theatre (results are averaged on 10 measurements).

AIR and Surface Analysed	Sabouraud Pre-Treatment	Sabouraud Post Treatment	(PCA) Pre-Treatment	(PCA) Post Treatment
AIR (CFU/m^3^)	0	0	5	1
DESK (CFU/plate)	0	0	2	0
TABLE (CFU/plate)	0	0	0	0
Furniture (CFU/plate)	0	0	1	0
